# Magnetic Nanofibrous Scaffolds Accelerate the Regeneration of Muscle Tissue in Combination with Extra Magnetic Fields

**DOI:** 10.3390/ijms23084440

**Published:** 2022-04-18

**Authors:** Xuechun Hu, Wenhao Liu, Lihong Sun, Shilin Xu, Tao Wang, Jie Meng, Tao Wen, Qingqiao Liu, Jian Liu, Haiyan Xu

**Affiliations:** 1 Institute of Basic Medical Sciences, Chinese Academy of Medical Sciences & Peking Union Medical College, Beijing 100005, China; huxuechun@ibms.pumc.edu.cn (X.H.); bo163169@163.com (L.S.); xushilin@ibms.pumc.edu.cn (S.X.); wangtao940215@126.com (T.W.); mengjie@ibms.pumc.edu.cn (J.M.); went@ibms.pumc.edu.cn (T.W.); liuqingqiao@ibms.pumc.edu.cn (Q.L.); 2 Peking Union Medical College, Beijing 100073, China; wh-liu16@mails.tsinghua.edu.cn

**Keywords:** magnetic scaffold, skeletal muscle, regeneration, magnetic field, angiogenesis, differentiation

## Abstract

The reversal of loss of the critical size of skeletal muscle is urgently required using biomaterial scaffolds to guide tissue regeneration. In this work, coaxial electrospun magnetic nanofibrous scaffolds were fabricated, with gelatin (Gel) as the shell of the fiber and polyurethane (PU) as the core. Iron oxide nanoparticles (Mag) of 10 nm diameter were added to the shell and core layer. Myoblast cells (C2C12) were cultured on the magnetic scaffolds and exposed to the applied magnetic fields. A mouse model of skeletal muscle injury was used to evaluate the repair guided by the scaffolds under the magnetic fields. It was shown that VEGF secretion and MyoG expression for the myoblast cells grown on the magnetic scaffolds under the magnetic fields were significantly increased, while, the gene expression of *Myh4* was up-regulated. Results from an in vivo study indicated that the process of skeletal muscle regeneration in the mouse muscle injury model was accelerated by using the magnetic actuated strategy, which was verified by histochemical analysis, immunofluorescence staining of CD31, electrophysiological measurement and ultrasound imaging. In conclusion, the integration of a magnetic scaffold combined with the extra magnetic fields enhanced myoblast differentiation and VEGF secretion and accelerated the defect repair of skeletal muscle in situ.

## 1. Introduction

Skeletal muscle plays a crucial role in the human body, controlling force generation and motor activity, and comprising approximately 40% of body weight [1,2]. Traumatic accidents, tumor resections, and degenerative diseases lead to great skeletal muscle loss. Although human skeletal muscle has a robust regenerative capacity, the severe muscle defects disrupt the muscle regenerative response. In general, with the loss of more than 20% of the muscle mass, the endogenic regeneration process fails and leads to scar tissue formation and loss of function [2,3]. The most widely used treatment for severe muscle tissue injury is surgical reconstruction [4]. Unfortunately, the low survival rate and the deficiency of donor sources severely limit its application [3,5], and challenges of heavy financial burden and limited therapeutic approaches are faced [1,4].

Tissue engineering is expected to provide alternative ways to promote skeletal muscle regeneration in which biomaterial scaffolds play roles in establishing microenvironments and providing physical and biochemical cues to different kinds of cells in the defect area to guide the in situ regeneration of the skeletal muscle and promote the repair process [6]. It is vital for biomaterial scaffolds in regenerative medicine to mimic the in vivo environment of cells and enhance the cells’ activity [7]. For example, appropriate scaffolds can bridge the gap between in vitro and in vivo cell culture, promoting drug research by cancer tissue engineering [8]. To enhance the tissue regeneration of bone, skin, and brain, polymeric scaffolds are required to have essential characteristics to meet the bioactivity and mechanical properties of the defective or damaged tissues to achieve repair or replacement [9,10,11].

It has been known that mechanical stimulation generated by a bioreactor can activate the proliferation and differentiation of myoblasts and make myoblasts fuse into myotubes [12]; however, it is challenging to apply mechanical stimulations to cells growing in the implanted scaffolds. Magnetic force has been demonstrated to have impacts on the cell cytoskeleton to facilitate skeletal muscle regeneration. For example, the stimulation generated by magnetic fields can increase myogenic differentiation and myotube orientation and induce myogenic cells to align in parallel bundles [13,14]. Moreover, magnetic fields are reported to prevent excess reactive oxidative stress to facilitate the regeneration process of skeletal muscle and minimize inflammatory infiltration and formation of scars after trauma [15,16]. In recent years, increasing investigations have indicated that magnetic scaffolds can transfer the force of magnetic fields efficiently to mechanical stimulations towards cells growing in the scaffolds, which can accelerate tissue regeneration [17,18]. Our previous research showed that the scaffolds with superparamagnetic property enhanced osteogenesis under the extra applied magnetic fields [19,20]. Other research has shown that a combination of superparamagnetic scaffolds and magnetic fields can promote the tissue regeneration of bone [21,22,23,24,25,26,27,28,29], cartilage [30,31], vessel [32,33,34], nerve [35,36,37], tendon [38], and play positive roles in the regulation of wound healing phenotypes for macrophages and fibroblasts [39,40].

Inspired by the magnetic actuating effects on biomaterial scaffold-guided tissue regeneration, in this work we fabricated a coaxial electrospun superparamagnetic nanofibrous scaffold and investigated magnetic actuating effects on the regeneration of skeletal muscle in vitro and in vivo. In the electrospun fibers, polyurethane (PU) was selected as the core because it has excellent thermoplastic mechanical property and is a promising candidate for use in the scaffolds of skeletal muscle, as muscle is a classical soft tissue and often has to stretch and contract. Meanwhile, in order to enhance cell adhesion to the scaffolds, gelatin (Gel) was selected as the shell content. We showed that the magnetic nanofibrous scaffold combined with the extra magnetic fields significantly enhanced the VEGF secretion and the differentiation of myoblast cells, which accelerated the process of skeletal muscle regeneration in a mouse model of muscle injury.

## 2. Results

### 2.1. Fabrication and Characterization of the Coaxial Electrospun Scaffolds

**Figure 1:**
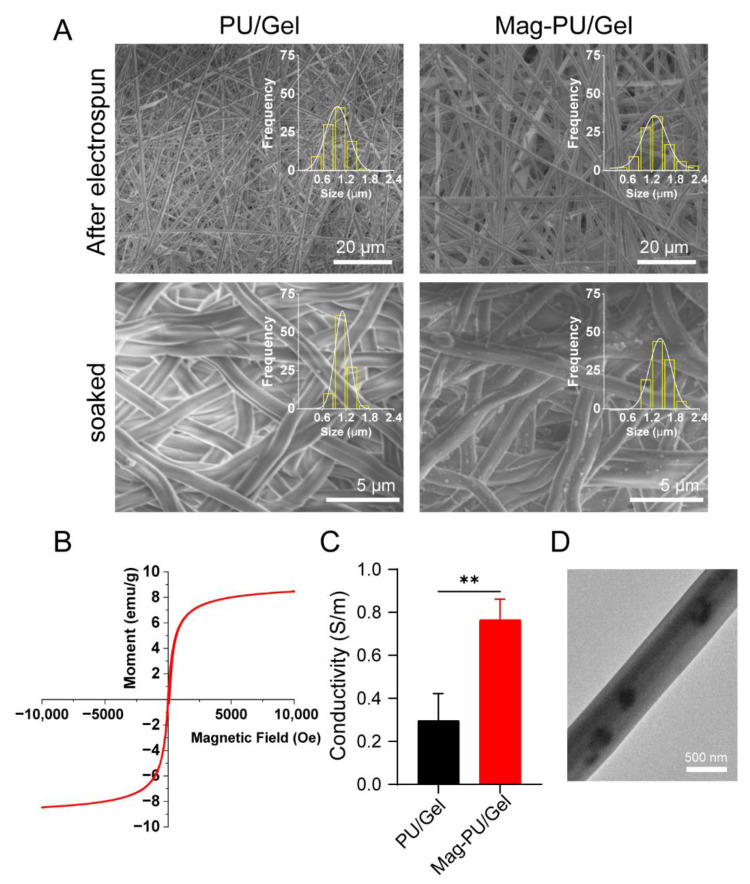
Characterizations of electrospun superparamagnetic fibrous scaffolds. (**A**) SEM images of different scaffolds. (**B**) Magnetization curve of Mag-PU/Gel. (**C**) Conductivity of different scaffolds. (**D**) Representative TEM image of the nanofibers of Mag-PU/Gel. These data were analyzed with two-tailed Student’s *t*-test: ** *p* < 0.01. * indicated significant difference.

By using the coaxial electrospinning technique, two kinds of composite scaffolds were fabricated, referred as Mag-PU/Gel and PU/Gel. The reason for selecting gelatin (Gel) and polyurethane (PU) was that Gel is a biological material with excellent biocompatibility and is cell-adhesive, and PU is a thermal elastic polymer that can provide both strength and elasticity to the electrospun scaffolds, as well as biocompatibility. Observations from scanning electron microscopy (SEM) indicated that there were randomly entangled nanofibers forming fibrous networks in the scaffold (Figure 1A top). By randomly counting and measuring 100 fibers, the average diameter of PU/Gel was 966.69 ± 256.72 nm, and that of Mag-PU/Gel was 1310.56 ± 253.51 nm (inserted in Figure 1A top). The addition of Fe_3_O_4_ nanoparticles (Fe_3_O_4_ NPs) caused the diameter of the fiber to increase slightly but had no significant effect on the morphology of the fiber and the network structure. After soaking in deionized water, the fibers of both PU/Gel and Mag-PU/Gel became slightly thicker (Figure 1A bottom). Importantly, Mag-PU/Gel exhibited superparamagnetic property under the applied magnetic fields; the saturated magnetization value was 6.36 emu/g (Figure 1B). Images from transmission electron microscopy (TEM) showed that the nanofibers had a core-shell structure. In Figure 1D the core is shown as dark grey and the shell as light grey, with black Fe_3_O_4_ NPs distributed in both core and shell. Moreover, the incorporation of Fe_3_O_4_ NPs brought a conductive property to Mag-PU/Gel, in that the conductivity was 0.77 S/m in PBS buffer and the conductivity value was twice that of PU/Gel (0.30 S/m) in the same buffer (Figure 1C). This would be beneficial to tissue regeneration because electrical signals are critical physiological stimuli modulating the regeneration process of muscles, nerves, and bones [41]. In particular, conductive biomaterials can strengthen intercellular coupling and realize synchronous contraction of muscle tissue [42]. The Zeta potential of Fe_3_O_4_ NPs was measured as −33.73 ± 0.54 mV, and the Zeta potential of the suspension of Mag-Gel was 26.32 ± 1.73 mV. This is because the isoelectric point of the Gel is about 7.0–9.5; the Gel was positively charged in the aqueous suspension. Therefore, it was rational to infer that the surface of the Mag-PU/Gel fibers was positively charged, which would be beneficial to cell adhesion [43]

### 2.2. Composite Scaffolds Supported Cells Growth under the Magnetic Fields

**Figure 2:**
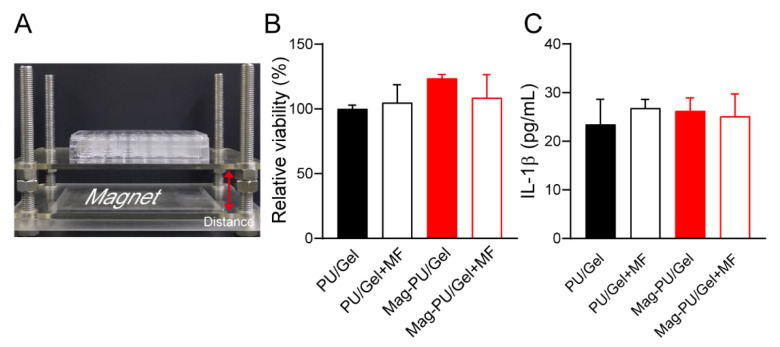
Viability and inflammatory effect of cells cultured under different conditions. (**A**) The cell culture device with magnetic fields. (**B**) The viability of C2C12 cells under different culture conditions. (**C**) The IL-1β secretion of RAW 264.7 cells incubated with the different scaffolds and magnetic field.

To evaluate the effect of the superparamagnetic property on the viability of C2C12 myoblast cells, a cell culture system with magnetic fields was established. The strength of the magnetic fields was set as 16 mT by adjusting the distance between the culture plate and the magnet (Figure 2A). Four groups were set in the experiment, including magnetic scaffold under magnetic fields (referred as Mag-PU/Gel + MF), magnetic scaffold without magnetic fields (Mag-PU/Gel), and the corresponding groups PU/Gel + MF and PU/Gel. Results showed that no difference was detected in the viability of the cells grown on the tested scaffolds (Figure 2B), indicating that the magnetic composite scaffold was able to support the growth of the cells, and the stimulation of magnetic fields had no significant effects on the viability of the cells. Furthermore, the inflammation response of RAW 264.7 cells to the different scaffolds and the magnetic field was assessed by measuring IL-1β secretion. It was shown that there was no significant difference in the production of IL-1β in the tested groups (Figure 2C), suggesting that the extra magnetic fields and the magnetic scaffold did not increase the potential of inflammatory responses more than PU/Gel. In addition, our previous investigation showed that polylactic acid based superparamagnetic scaffolds with magnetic fields were able to drive RAW264.7 cells to polarize towards an M2-like phenotype [44], suggesting the strategy of magnetic scaffolds combined with magnetic fields does not exert significant inflammatory responses.

### 2.3. Magnetic Scaffolds Enhanced Cell Differentiation under the Magnetic Fields

When the skeletal muscle is injured, satellite cells begin to migrate toward the injury site and reenter the cell cycle to proliferate, and then myoblasts exit the cell cycle and differentiate into mature myocytes [45]. Therefore, we next investigated whether the magnetic scaffold was beneficial to the maturation of C2C12 cells.

**Figure 3:**
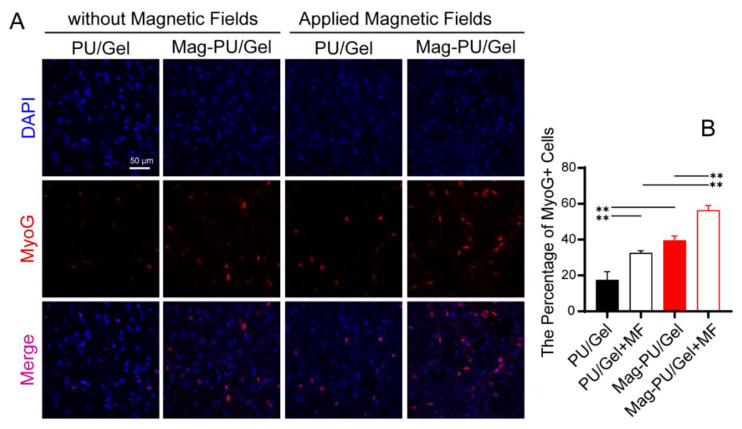
Effects of magnetic scaffolds and magnetic fields on the expression of MyoG. (**A**) Immunofluorescence staining of MyoG protein in C2C12 cells after incubation under different conditions for 7 days, MyoG positive cells were stained as red, and nuclei were stained as blue. (**B**) Percentage of MyoG positive cells in different groups. These data were analyzed by one-way analysis of variance with Tukey’s post hoc test: ** *p* < 0.01. * indicated significant difference.

**Figure 4:**
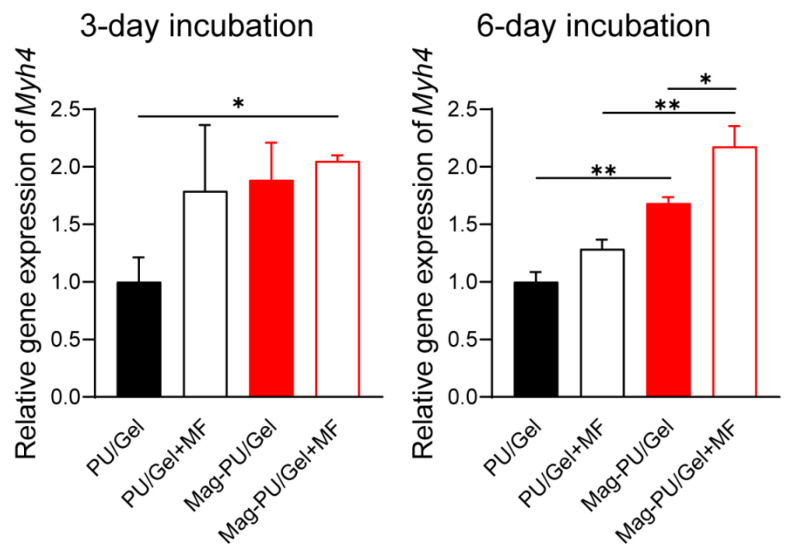
Gene expression of *Myh4* in C2C12 cells grown on different scaffolds for 3 days and 6 days with and without the applied magnetic fields. These data were analyzed by one-way analysis of variance with Tukey’s post hoc test: * *p* < 0.05, ** *p* < 0.01. * indicated significant difference.

Myogenin (MyoG) is an indicative protein appearing in the middle and late-stage maturation of myoblasts [46]. Herein, the expression of MyoG was examined by using laser confocal microscopy. It was seen that there were much more MyoG positive cells in the group of Mag-PU/Gel + MF after 7-day incubation in the differentiation medium than in the other groups, followed by Mag-PU/Gel, PU/Gel + MF, and PU/Gel. In particular, there were only scattered MyoG positive cells on the PU/Gel + MF and PU/Gel (Figure 3). To further confirm the maturation of myoblasts, the gene expression of *Myh4*, an important indicator of the myosin heavy chain appearing in the late stage of the maturation [47,48], was examined after 3-day and 6-day incubation (Figure 4). It was shown that after the 6-day incubation cells in the PU/Gel + MF group reached the highest level of *Myh4*. These results suggested that the magnetic scaffold combined with the extra applied magnetic fields was able to promote the differentiation of C2C12 cells.

### 2.4. Magnetic Scaffolds Combined with the Magnetic Fields Promoted VEGF Production

**Figure 5:**
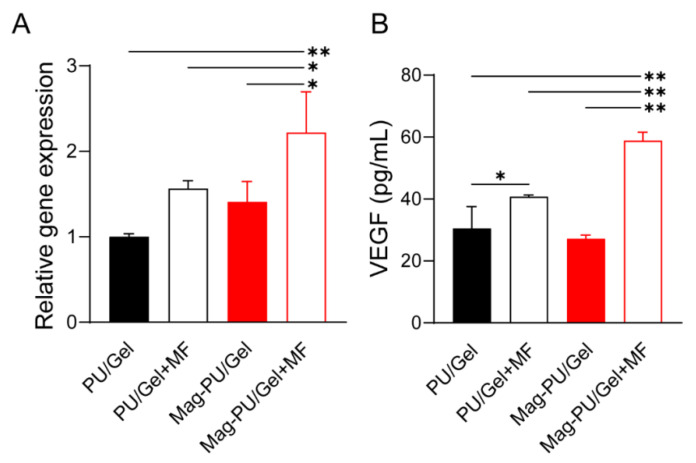
Effects of magnetic scaffolds combined with the magnetic fields on VEGF production of C2C12 cells. (**A**). The relative gene expression of *Vegfa* normalized to PU/Gel group (*Gapdh* served as reference). (**B**). Secretion of VEGF detected by ELISA. These data were analyzed by one-way analysis of variance with Tukey’s post hoc test: * *p* < 0.05, ** *p* < 0.01. * indicated significant difference.

In the process of muscle regeneration, it is crucial to maintain sufficient oxygen diffusion in the defect area because of the high metabolic rate of skeletal muscle, which is largely dependent on the angiogenesis of the area [49]. Herein, we investigated VEGF secretion as well as the gene expression of *Vegfa* in C2C12 myoblast cells grown on different scaffolds (Figure 5). It was shown that no significant difference was detected in the *Vegfa* expression among the cells cultured on PU/Gel, PU/Gel + MF, and Mag-PU/Gel, while the *Vegfa* expression of the cells on Mag-PU/Gel + MF was significantly higher than that on the other groups, being approximately 2.2-times higher than that on PU/Gel and 1.5-times higher than that on PU/Gel + MF and Mag-PU/Gel. Moreover, the secretion of VEGF by the cells on scaffolds with applied magnetic fields was significantly higher than that without the fields, no matter the PU/Gel or Mag-PU/Gel, but the magnetic scaffold played additional positive roles, as shown by secreted VEGF in the Mag-PU/Gel + MF group being approximately 2.2 times of that without magnetic fields, only about 1.3-times in the PU/Gel + MF and PU/Gel groups.

### 2.5. Magnetically Actuated Scaffold Accelerated the Regeneration of Muscle Tissue

**Figure 6:**
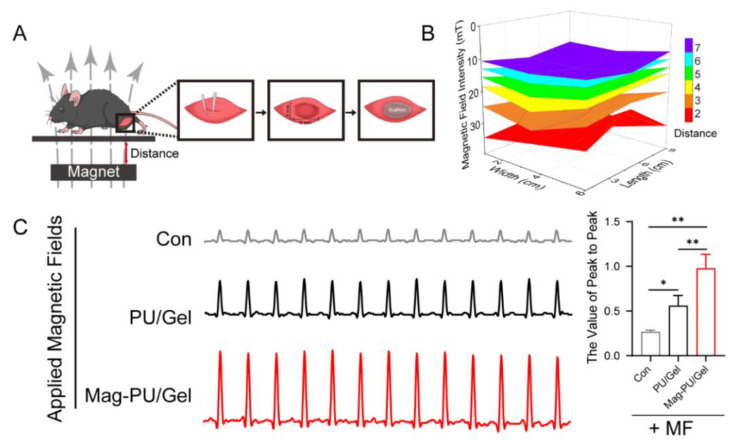
Effects of magnetic scaffolds combined with magnetic fields on the recovery of the electrophysiological function of the skeletal muscle. (**A**) Schematic diagram of animal experiments. (**B**) Strength distribution of magnetic fields in the cages for mice. (**C**) Muscle contraction tension curve of different groups in response to the electrical stimulation. These data were analyzed by one-way analysis of variance with Tukey’s post hoc test: * *p* < 0.05, ** *p* < 0.01. * indicated significant difference.

The effect of the magnetic scaffolds on skeletal muscle regeneration was investigated in a volumetric muscle loss injury mouse model in which a piece of muscle tissue (6 × 5 × 2 mm) was cut off and the tested scaffold was implanted in the defect site (Figure 6A). The mice were fed in cages with a magnet on the bottom. The strength distribution of the magnetic fields in the middle of the cage was measured as shown in Figure 6B, and the mice were located in the area of 13–20 mT of the fields. After 20-day implantation, the muscle contraction tension curve of the implanted area to electrical stimulation was recorded by a biological signal acquisition and analysis system (Figure 6C). Compared with the non-implantation (control + MF) and PU/Gel + MF groups, the muscle of mice in the Mag-PU/Gel + MF groups generated stronger contraction. The contractile force-generating capacity of muscle in the Mag-PU/Gel + MF group was approximately twice that in the PU/Gel + MF group, and was four times of the group of the non-implantation control, which clearly indicates that Mag-PU/Gel + MF could effectively improve the recovery of the function of the skeletal muscle.

**Figure 7:**
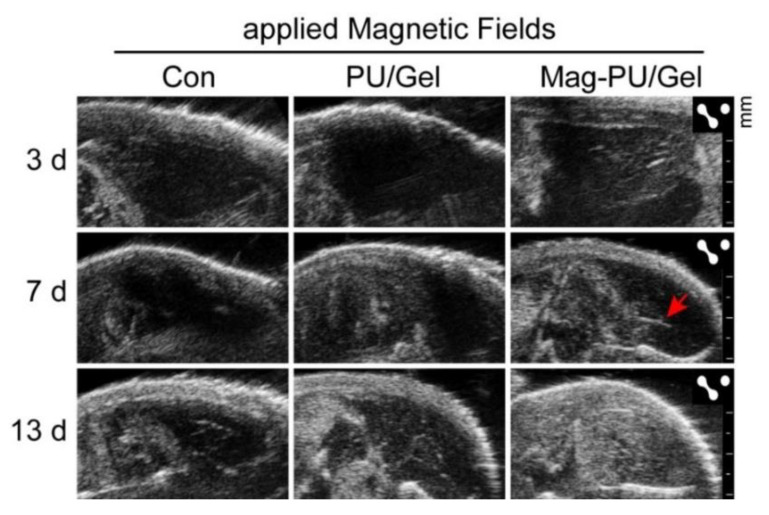
Ultrasonic imaging of the defected area of mice skeletal muscle on different days after the scaffold implantation.

To verify tissue regeneration guided by the implanted scaffolds, ultrasonic imaging was used to observe the newly formed tissue and blood supply of the defect section (Figure 7). In the ultrasonic images, the dark area represented the tissue defect where echo signals were unable to be detected. Thus, the image with the largest dark area was selected for comparison. When the postoperative congestion disappeared, defects were observed clearly on the third day after implantation, which was similar in the three groups. It was noted that there was new tissue observed in the lacerated regions in the Mag-PU/Gel + MF group on day 7, while more muscle losses remained at the defect sites in the non-implantation control and PU/Gel + MF groups. It was noticed that after 13 days of implantation, there were more newborn tissues and significant volume recovery in the Mag-PU/Gel + MF group, in which the defect site was filled with well-regenerated tissue with a clear fibrous structure. Moreover, the significant role of the Mag-PU/Gel scaffold combined with the magnetic fields in angiogenesis could be seen in the images; after 7 days, the complete vascular structure had formed only in the Mag-PU/Gel + MF group (indicated by the red arrow).

**Figure 8:**
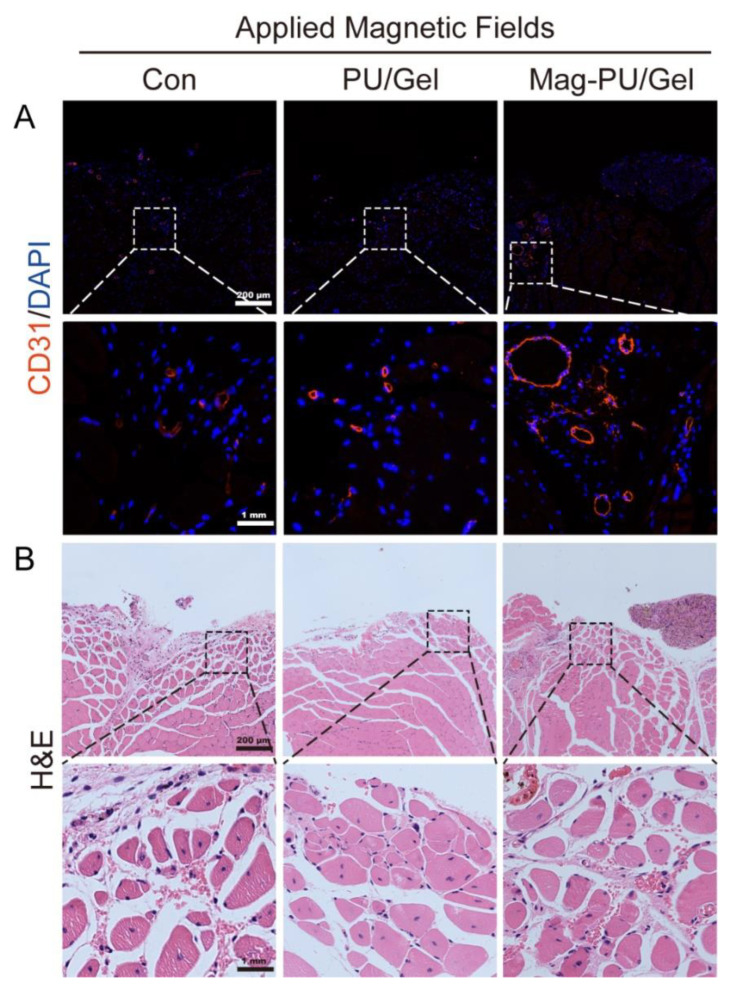
Effects of magnetic scaffolds combined with magnetic fields on tissue regeneration in situ after scaffold implantation. (**A**) Immunological fluorescent staining of CD31. (**B**) H&E staining of tissue in the defect area. The bottom panel in (**A**,**B**) shows magnified images within the dash-lined rectangle.

To confirm the formation of vascular structure, the immunofluorescence staining of CD31, as well as Hematoxylin-Eosin (H&E) Staining, were performed. As shown in Figure 8A, CD31 positive cells (labeled by red fluorescence) in the Mag-PU/Gel + MF group were more prevalent than that in the non-implantation control + MF and PU/Gel + MF groups, indicating there were more vascular structures formed in the defect area in the Mag-PU/Gel + MF group than in the other groups. Moreover, in the magnified images, the newly generated vascular structures in the Mag-PU/Gel + MF group were not only located at the scaffolds-implanted site but also around the muscle fibers, which are important to maintain the mechanical properties of regenerated muscle tissues [50]. These observations support the results of VEGF production in vitro.

Myofibers with nuclei in the center of the cytoplasm are usually considered newborn myofibers, while mature ones with nuclei are on the periphery [51]. The H&E staining of the defect sections showed that there were evident newborn tissues in the Mag-PU/Gel + MF group, in which the lacerated region almost disappeared, and the defect site was filled with newborn muscle fibers and connective tissues. The myofibers in the Mag-PU/Gel + MF group seemed finer and closer than those in the non-implantation control + MF group, while the cross-section shape of the myofibers in the Mag-PU/Gel + MF group was more homogeneous than that in the PU/Gel + MF group (Figure 8B). These results provide more evidence that the magnetic scaffolds combined with the magnetic fields were able to accelerate the process of skeletal muscle repair.

## 3. Discussion

As an important part of the human body, skeletal muscle tissue is easily damaged by toxic chemicals, biological factors, or improper exercise, and the loss of significant muscle tissue seriously affects the life quality of patients. There have been many achievements in the development of new ways for guiding muscle tissue regeneration using polyurethane or gelatin, or a combination of both, as scaffolds. For example, gelatin-genipin-based biomaterials [52], gelatin/alginate composites [53] and the composite of gelatin/polycaprolactone [54] have been used for skeletal muscle tissue engineering. Polyurethane-based biodegradable elastomer is also a promising material for skeletal muscle tissue engineering by using casting or electrospinning in combination with 3D printed polycaprolactone [55,56].

Although it is well known that mechanical stimulation can activate the proliferation and differentiation of myoblasts and make myoblasts fuse into myotubes [12,57], how to apply mechanical stimulation to the implanted scaffolds, and the stimulation transferred to the regenerative cells, is a crucial issue. In this work, we utilized the superparamagnetic performance of the magnetic scaffolds to transfer a magnetic force for the mechanical stimulation of the cells in vitro and in vivo. Using this approach, the regeneration process of the skeletal muscle tissue was accelerated, indicating an effective way to promote the defect repair of the skeletal muscle. PU was used as the core of the electrospun fibers, providing excellent elasticity and strength; however, the currently used PU is not biodegradable. Our main goal was to proof of concept in this research, and biodegradable PU will be used in the magnetic scaffolds in the future. It is well documented that the degradation of gelatin depends on the crosslink process and, based on similar crosslink processes described in the literature [58], the Gel in the shell may not be largely degraded within 2 days in the culture medium.

In addition to promoting the differentiation of the myoblast cells, the strategy of magnetic scaffolds in combination with magnetic fields significantly increased VEGF production. Due to nonspecific adsorption of proteins to the scaffolds, it was very hard to collect cell lysates completely to perform Western blotting for CD31, but VEGF production was determined. The results obtained from in vivo experiments were consistent with the increased VEGF production in vitro, and there was clear evidence that more vascular structures were formed in the Mag-PU/Gel + MF group (Figure 7 and Figure 8). These results indicated that the angiogenesis function of myoblasts could be strongly enhanced by the use of magnetic actuation, and the newly formed blood vessels could provide a constant oxygen supply for muscle regeneration. It is well documented that angiogenesis can promote skeletal muscle regeneration by enhancing the migration, proliferation, and myogenesis differentiation of myogenic progenitor cells [59]. Therefore, the promoted VEGF production was believed to be very important and beneficial for the resulting tissue regeneration.

## 4. Materials and Methods

### 4.1. Preparation of Scaffolds

Iron oxide nanoparticles modified by 2, 3-dimercaptosuccinic acid (DMSA) with a core size of 10 nm (Fe_3_O_4_ NPs) were purchased from Nanjing Nanoeast Biotech Co., LTD (Nanjing, China). Gelatin (Gel) type A from porcine skin was purchased from Sigma-Aldrich (V9000863, Waltham, MA, USA) with an average molecular weight of 50–100 kDa and an isoelectric point of 7.0–9.5. To prepare magnetic scaffolds, 0.2 g of Fe_3_O_4_ NPs was dispersed in 10 mL of Hexafluoroisopropanol (HFIP) using an ultrasonic probe for 10 min to form a homogenous suspension. Then, 1 g of Gel or 1 g of polyurethane (PU, YR-80P, Yantai Wanhua Polyurethane Co., Ltd., Yantai, China) was dissolved in the suspension. The prepared suspension was referred as Mag-Gel or Mag-PU, respectively. The former was used as the shell component and the latter was used as the core component. Both were loaded into 5 mL syringes equipped with a coaxial electrospinning device. The distance between the needle and the collector was 15 cm, and the voltage was 20 kV. The flow rate of the suspension and the spinning time were fixed at 0.0020 mm/s and 2 h, respectively. Electrospinning was performed at room temperature and a humidity of 50%. The collected film (referred as Mag-PU/Gel) was vacuum dried for 48 h followed by cross-linking at 120 °C for 4 h. As a control, a film composed of fibers with the shell of Gel alone and a core of PU alone (referred as PU/Gel) was prepared using the same procedure.

### 4.2. Physicochemical Characterizations

The morphology of PU/Gel and Mag-PU/Gel were examined by scanning electron microscopy (SEM, SU-8010, Hitachi, Ibaraki, Japan). The average diameter of fibers was determined by randomly counting and measuring 100 fibers in the SEM images using Image J software (NIH, Bethesda, MD, USA). The core-shell structure of the fibers and the distribution of Fe_3_O_4_ NPs in Mag-PU/Gel were observed by transmission electron microscopy (TEM, HT7700, Hitachi, Ibaraki, Japan). The superparamagnetic property of Mag-PU/Gel was measured by a vibrating sample magnetometer (VSM, Lakeshore 7407, Columbus, OH, USA), and the conductivity was measured using the four-point probe technique (Loresta-GX MCP-T700, Mitsubishi Chemical, Tokyo, Japan). The Zeta potential of Fe_3_O_4_ NPs and the suspension of Fe_3_O_4_ NPs and Gel (Mag-Gel) were measured using dynamic laser scattering (DLS, NanoBrook Omni, Brookhaven, New York, NY, USA).

### 4.3. Cell Culture

C2C12 myoblast cells and RAW 264.7 were purchased from the Cell Center of the Institute of Basic Medical Sciences, Chinese Academy of Medical Sciences (Beijing, China). The cells were cultured in Dulbecco’s Modified Eagle’s Medium (DMEM, Hyclone, Logan, UT, USA) supplemented with 10% FBS and 1% penicillin-streptomycin (p/s) under standard culture conditions (37 °C, 5% CO_2_). The scaffolds were trimmed to an appropriate diameter fitting that of the culture wells and sterilized with UV irradiation following soaking in 75% ethanol. The C2C12 cells were seeded on the scaffolds and cultured in standard medium overnight followed by replaced with the differentiation medium (DMEM supplemented with 2% horse serum and 1% p/s), and then cultured for additional days.

### 4.4. Setup of Magnetic Fields for the Cell Culture System

The device providing magnetic fields for the cell culture was built by fixing a permanent magnet beneath the cell culture plate. The strength of magnetic fields was adjusted by changing the distance between the magnet and the culture plate and measured by a Teslameter (SG-4L, Zhuoshengjia Electromagnetic Technology, Beijing, China). The strength of magnetic fields was set at 16 mT and the fields were applied at intervals of 12 h during the culture.

### 4.5. Cell Viability Assay

C2C12 cells (1 × 10^4^) were seeded in a 96-well plate with trimmed PU/Gel or Mag-PU/Gel scaffolds in the wells. The plate was placed on a device that provided magnetic fields at 12 h-intervals during the culture. Controls without the magnetic fields were set. After a 2-day incubation, 100 μL of culture medium and 10 μL of Cell Counting Kit 8 reagent (CCK-8, Dojindo, Kumamoto, Japan) were added to each well followed by incubation for 2 h at 37 °C. The supernatants were transferred to a microplate to measure the absorbance at 450 nm by a microplate reader (Synergy H1, Bio Tek, Winooski, VT, USA).

### 4.6. Immunofluorescence Staining Assay

C2C12 cells (5 × 10^4^) were seeded on 24-well culture plates with trimmed PU/Gel and Mag-PU/Gel fixed at the bottom of the wells by glass rings, and a 16 mT magnetic field was applied to the plate alternately at intervals of 12 h. After a 7-day incubation with differentiation medium, the cells grown on the different scaffolds were immunostained for Myogenin. The cells were fixed by 4% paraformaldehyde in PBS and permeabilized in 0.5% Triton X-100 in PBS. After that, PBS with 5% FBS was used to block the non-specific binding sites, and they were sequentially incubated with 1 μg/mL Anti-Myogenin antibody (Abcam, ab1835, Cambridge, UK) overnight at 4 °C, and then incubated with Alexa Fluor^®^ 594 conjugated goat anti-mouse IgG (1:1000, CST, Boston, MA, USA). Nuclear DNA was labeled by DAPI in the mounting medium. For each group, three replicates were made and the images were captured using a laser confocal microscope (Leica SP8 STED, Leica, Wetzlar, Germany).

### 4.7. Quantitative Real-Time PCR (qRT-PCR) Analysis Assay

C2C12 cells (2 × 10^5^) were seeded on 24-well culture plates with trimmed PU/Gel and Mag-PU/Gel fixed at the bottom of the wells by glass rings, and a 16 mT magnetic field was applied to the plate alternately at intervals of 12 h. Total RNA of cells after a 3-day incubation and 6-day incubation with differentiation medium was extracted and the concentration was determined using a UV-Vis Spectrophotometer (NanoDrop 1000, ThermoFisher Scientific, Waltham, MA, USA). The RNA was then reverse transcripted into cDNA using PrimeScript^TM^ RT Master Mix (Takara Bio, Shiga, Japan), which was used as templates for qRT-PCR. qRT-PCR was performed using a SRBY Green probe with a QuantStudio 1 Real-Time PCR System (ThermoFisher Scientific, 40 cycles, melting at 95 °C for 15 s, annealing and extension at 60 °C for 30 s). The gene expression of *Vegfa* was detected after 3 days of incubation, while the expression levels of *Myh4* were detected after three and six days of incubation. The expression level of each gene was analyzed by the comparative threshold cycle (ΔCt) method and *Gapdh* served as the reference. The expression of each gene was normalized by PU/Gel group. The sequences of the oligonucleotide primers were as follows.

*Myh4* forward primer, 5′-TTGAAAAGACGAAGCAGCGAC-3′,
*Myh4* reverse primer, 5′-AGAGAGCGGGACTCCTTCTG-3′,
*Vegfa* forward primer, 5′-GCACATAGAGAGAATGAGCTTCC-3′,
*Vegfa* reverse primer, 5′-CTCCGCTCTGAACAAGGCT-3′,
*Gapdh* forward primer, 5′-TGACCTCAACTACATGGTCTACA-3′,
*Gapdh* reverse primer, 5′-CTTCCCATTCTCGGCCTTG-3′.

### 4.8. Cytokines Production Assay

C2C12 cells (5 × 10^4^) were seeded in 24-well culture plates with trimmed PU/Gel and Mag-PU/Gel fixed at the bottom of the wells by glass rings, and a 16 mT magnetic field was applied to the plate alternately at intervals of 12 h. After incubation for 3 days, the culture supernatants were collected to determine the production of mouse VEGF by an enzyme-linked immunosorbent assay (ELISA) kit (Neobioscience, Guangdong, China). RAW 264.7 cells (4 × 10^5^) were seeded and cultured using the same protocol as the C2C12cells. After a 3-day incubation, the supernatants were collected to detect the secretion of IL-1β by an ELISA kit (Neobioscience, Guangdong, China). The brief procedure of ELISA was as follows. Supernatants of 100 μL were incubated at 37 °C for 90 min with the plate pre-coated with antibodies. After the supernatant was removed, the plate was washed and incubated with biotinylated antibodies at 37 °C for 60 min. Then, horseradish peroxidase (HRP) linked streptavidin solution and the Tetramethylbenzidine (TMB) solution were added sequentially and incubated for 30 min and 15 min, respectively. The absorbance at 450 nm was read by a microplate reader within 3 min of adding the stop solution. A standard curve was made to calculate the concentration of the cytokines in the supernatants.

### 4.9. Mice Skeletal Muscle Injury Model

To build the tibialis anterior muscle defect model, eight-week-old male C57BL/6 mice weighing between 20–25 g were employed. All mice were randomly divided into three groups: a blank control group, a PU/Gel group, and a Mag-PU/Gel group. All mice were acclimatized for 1 week before surgery. The mice were anesthetized by an intraperitoneal injection of 10% urethane in PBS. After disinfection by iodophor and 75% ethanol, an anterolateral skin incision was made to expose the tibialis anterior muscle. A defect of about 30–50% of the total tibialis anterior muscle was created on the muscle belly. For scaffold implantation groups, 6 mm diameters of the PU/Gel scaffold and the Mag-PU/Gel scaffold were fitted on the wound of the respective groups. The control group underwent a sham operation without any treatment. The incision was then sutured using absorbable surgical sutures. After waking up, the mice were put into cages beneath which the magnets were placed to produce magnetic fields of 13–20 mT. The animal experiment was approved by the committee on the Animal Care and Use of the Institute of Basic Medical Sciences, Chinese Academy of Medical Sciences.

### 4.10. Electrophysiological Signal Measurement

After implantation for 20 days, an incision was made on the anesthetized animal to expose the posterior thigh muscle of scaffolds in the implanted limb. Along the crack between the biceps femoris and semi-membranous muscle, the sciatic nerve was separated by a glass needle. A stimulation electrode was placed on the sciatic nerve and a tension transducer was connected to the ankle. Electrical stimulation was applied with an intensity of 2 V, a duration of 200 ms, and a frequency of 1 Hz. The muscle contraction tension curve was recorded by a biological signal acquisition and analysis system (BL-420s, Techman, Chengdu, China) when the wave was stable.

### 4.11. Ultrasound Imaging Assay

Mice were subjected to high-resolution ultrasound scan imaging on days 3, 7, and 13 post-implantation using the Vevo 2100 small animal ultrasound system (FUJIFILM VisualSonics Inc., Toronto, ON, Canada) with the MS-700 transducer at a center frequency of 50 MHz.

### 4.12. Hematoxylin-Eosin (H&E) Staining and Immunofluorescence Staining

Muscle samples were collected on day 20 after implantation and fixed with 4% paraformaldehyde. The tissues were then cut into paraffin sections (thickness, 3–4 μm) and stained with Hematoxylin-Eosin following standard protocols. For immunofluorescence staining, the sections were deparaffinized and antigen retrieval was performed. The sections were incubated with CD31 primary antibody overnight at 4 °C, washed in PBS, and incubated in Cy3 labeled goat anti-rabbit IgG secondary antibody for 50 min at room temperature, and finally mounted by DAPI-contained mounting medium. Images were acquired using EVOS M7000 microscope (ThermoFisher Scientific, Waltham, MA, USA).

### 4.13. Statistical Analysis

Values were expressed as mean ± standard deviation. The significance between two groups was analyzed using two-tailed Student’s *t*-test. For multiple comparisons, one-way analysis of variance (ANOVA) with Tukey’s post hoc test was used. Statistical analysis was performed using GraphPad software (GraphPad 8.3.0, San Diego, CA, USA). Statistical difference was accepted at *p* < 0.05 and illustrated in the figures as *, *p* < 0.05 and **, *p* < 0.01.

## 5. Conclusions

We fabricated nanofibrous scaffolds by using a coaxial electrospinning technique. Fibers were composed of a polyurethane core and gelatin shell, and iron oxide nanoparticles integrated into the shell and core. The scaffolds had superparamagnetic responsive properties due to the addition of iron oxide nanoparticles. The magnetic responsive scaffold in combination with the extra applied magnetic fields enhanced the differentiation and VEGF secretion of the myoblast cell C2C12. In in vivo experiments, the repair of muscle tissue defects in mice was accelerated by implanting the magnetic responsive scaffold in situ and applying extra magnetic fields to the mice cages, evidenced by ultrasonic imaging of the defected area and recovery of the electrophysiological function of the skeletal muscle. It is suggested the strategy of using magnetic scaffold implantation combined with an extra applied magnetic field is a promising way to accelerate skeletal muscle tissue regeneration in situ.

## Data Availability

Not applicable.
